# Effectiveness of a new approach for exclusive breastfeeding counselling on breastfeeding prevalence, infant growth velocity and postpartum weight loss in overweight or obese women: protocol for a randomized controlled trial

**DOI:** 10.1186/s13006-019-0249-2

**Published:** 2020-01-06

**Authors:** Fanny Aldana-Parra, Gilma Olaya, Mary Fewtrell

**Affiliations:** 1 Departamento de Nutrición y Bioquímica, Pontificia Universidad Javeriana, Bogotá, Colombia; 2 UCL Great Ormond Street Institute of Child Health, London, UK

**Keywords:** Maternal obesity, Maternal overweight, Obesity prevention, Infant growth velocity, Prolactin in overweight women, Postpartum weight loss, Breastfeeding counselling, Exclusive breastfeeding, Breastfeeding randomized controlled trial, Centred-Client counselling on breastfeeding

## Abstract

**Background:**

Maternal overweight, infant feeding and early growth velocity are risk factors for obesity later in life. The first one thousand days are a window of opportunity to program health and disease. Exclusive breastfeeding may protect against obesity; however, it is not consistently practiced. Obesity rates have been increasing worldwide. Overweight or obese women have lower rates of breastfeeding and face mechanical, psychological and biological difficulties. Breastfeeding counselling is a successful strategy to support breastfeeding in normal weight women; but there is a lack of evidence on its effectiveness in overweight women. Our purpose is to evaluate a new approach to exclusive breastfeeding counselling based on Carl Rogers’ Centred-Client Theory in overweight women, and to examine effects on breastfeeding prevalence, infant growth velocity and maternal postpartum weight loss.

**Methods:**

A two-arm simple randomized controlled trial will be conducted in overweight and obese women recruited in a Baby Friendly Hospital in Bogotá, Colombia. The intervention is exclusive breastfeeding counselling based on Rogers’ theory but adapted for overweight women; it will be performed during the last month of pregnancy, 24 h after delivery and during early infancy (1 and 3 months postpartum). The primary outcomes will be exclusive breastfeeding prevalence, infant growth velocity and maternal weight loss from birth up to 4 months after delivery; and the secondary outcomes will be prolactin and macronutrient levels in breast milk and serum prolactin levels. Intention to treat analysis will be performed to estimate the effect of the new counselling approach compared to standard management on the prevalence of exclusive breastfeeding, infant growth velocity and maternal weight loss.

**Discussion:**

We hypothesize that the intervention will result in an increase in the initiation and maintenance of exclusive breastfeeding, allowing adequate infant growth velocity and maternal weight loss after delivery. It is hoped that the results of this trial will provide evidence to support public health policy on supporting breastfeeding in this vulnerable group of women.

**Trial registration:**

(UTN) **U1111–1228-9913** February 20th 2019; ISRCTN15922904

February 27th 2019, retrospectively registered.

## Background

In 2016, the World Health Organization (WHO) reported a worldwide prevalence of overweight (body mass index [BMI] higher than 25 kg/m^2^) and obesity (BMI > 30 kg/m^2^) of 40 and 15% respectively in women aged ≥18 years, with a global projection of 70 million overweight children aged under five years by 2025 [1], perpetuating the intergenerational transmission of obesity. In Colombia in 2015, the Instituto Colombiano de Bienestar Familiar (ICBF) reported a prevalence of overweight in the adult population of 37%, of which 22% were obese woman, and a prevalence of 6.3% of obesity in children under 5 years [2].

Several modifiable factors during the first thousand days (from conception to two years of age) are associated with obesity risk later in life including excess pre-pregnancy weight [3], excessive weight gain during pregnancy [4–6], infant sleep deprivation [7], formula feeding [8, 9], high infant protein intake [10] and lower duration of exclusive breastfeeding (EBF) [11]. The WHO recommends EBF until six months of age and breastfeeding with adequate complementary feeding up to 2 years of age or more [12–14]. However, the role of breastfeeding in obesity prevention remains controversial. Some studies have reported a lower risk of obesity in breastfed versus formula fed children at 42 months (AOR 0.78; 95% CI 0.7–0.85) [15], at school age (AOR = 0.75; 95% CI 0.57–0.98) [16] and during adolescence (OR = 3.37), after adjusting for sociodemographic factors and parental obesity [17]. In contrast, other studies have shown that an intervention to promote breastfeeding was not associated with infant BMI or triceps skinfold thickness at 6.5 years [18]; and the risk of overweight at 4 years [19] and 11 years was not related to total or predominant breastfeeding duration [20]. EBF during the first three months postpartum could improve maternal weight loss. Comparisons between breastfeeding versus formula feeding women showed a significant time-effect of breastfeeding on maternal weight loss between 2 and 24 months postpartum, with a mean 2 kg greater weight loss in breastfeeding mothers [21].

Despite the associated health benefits, only 43% of infants are exclusively breastfed during the first 6 months worldwide, and in 2015 this percentage was 36% in Colombia [22]. Breastfeeding initiation and duration are even lower in overweight women; those with pre-pregnancy BMI ≥ 30 kg/m^2^ are less likely to intend to exclusively breastfeed compared with normal weight and overweight women (78.8% vs 95.5 and 96.2%, respectively) [23]; whilst overweight women have significantly lower initiation and duration of breastfeeding than women in the normal range [24].

Low EBF and breastfeeding rates in overweight women [25] may be related to mechanical problems (congested mammary glands, larger amounts of adipose tissue, flattened areolas and oedema), delayed lactogenesis II as a consequence of inadequate suckling [26, 27], changes in prolactin levels [28], hypoplasia of the mammary gland and reduced stromal tissue [29]. Other risk factors are related to delayed early contact as a consequence of caesarean section [27] or a poor body image [30, 31]. Despite the reported lower rates of breastfeeding in obese mothers [30, there is some evidence that they are more likely to maintain EBF at 6 months than normal weight women if they are encouraged to exclusively breastfeed [32].

Interestingly, overweight women show lower serum prolactin and a lower prolactin response to suckling when compared with normal weight women at 48 h postpartum [26], which could explain the apparent delay of lactogenesis II and a shorter breastfeeding duration. Prolactin is involved in a variety of physiological processes in mammals [33] including the inhibition of lipogenesis [34] and an increase in glucokinase activity augmenting β cell secretion [35], resulting in an apparent inverse association between serum prolactin levels and metabolic syndrome and type 2 diabetes [36–38]. Prolactin in breast milk survives the infant gastrointestinal tract and is absorbed in the infant gut in a bioactive form or may act locally on the gut epithelium [39]; thus, maternal prolactin concentration could potentially influence infant fat and glucose metabolism during early lactation, both strongly related with obesity [40].

To date, only three experimental studies have been conducted using interventions that specifically aimed to prolong the duration of EBF until 6 months in obese mothers [41–43]. The strategies used included high intensity telephone support [43], peer counselling, breastfeeding education and anticipatory guidance [42], and low intensity telephone support with provision of a breast pump to facilitate lactation [41]. Only the intensive telephone intervention [43] resulted in prolonged EBF duration in obese mothers. The intervention had no effect on infant weight at 6 months and the EBF rate at 6 months remained lower than reported in the general Danish population [43]. It is possible that the support provided by telephone-based interventions is insufficient to improve breastfeeding practices in obese mothers. All studies were conducted in high-income countries, and no interventions have been developed or tested for low- or middle-income countries, including Colombia. Studies that aim to prolong breastfeeding and EBF in obese mothers should consider approaches that address the time-specific problems and the particular needs that these mothers face.

Since the middle of the twentieth century, counselling, defined as a “purposeful, private conversation arising from the intention of one person (couple or family) to reflect on and resolve a problem in their life, and the willingness of another person to assist in that endeavour” [44] has become one of the most widely used strategies for achieving health goals. It has been based on a wide variety of theoretical models and approaches, including the psychodynamic-interpersonal model [45], the cognitive-behavioural approach [46] and the person-centred approach [47]. In normal weight women, breastfeeding support based on counselling [48] by telephone, person to person, peer counselling and health care institution support strategies [49–51] have been shown to be effective for improving breastfeeding initiation, duration and exclusivity [52]. However, there is a lack of evidence regarding breastfeeding counselling specifically designed for overweight or obese women. Carl Rogers’ Client-Centred Theory allows the mother to take positive decisions towards a longer duration of breastfeeding and EBF as it enables the mother to recognize her strengths and limitations and those of her surroundings. Another important aspect of this theory is that it can be adapted to be used at critical time-points and to address specific problems faced by overweight mothers.

Considering the intergenerational transmission of obesity, the window of opportunity during the first thousand days, the positive effect of EBF counselling in normal weight women and the lack of evidence supporting an effective intervention to improve breastfeeding and EBF in overweight women, the purpose of this study is to evaluate the effects of a new approach to EBF counselling in overweight women, based on Carl Rogers´ Centred-Client Theory, on breastfeeding duration, infant growth velocity and maternal postpartum weight loss.

## Methods

### Study design

A two-arm randomized controlled trial was designed to identify the effect of a new EBF counselling intervention specifically designed to support overweight women, on the prevalence of breastfeeding and EBF, infant growth velocity and maternal postpartum weight loss up to 4 months. Although the primary end-point will be 4 months after delivery, mothers will be advised to continue EBF until 6 months, as recommended by WHO.

The trial involves overweight women recruited in the last month of pregnancy and their infants. Eligible overweight women will be randomly assigned to an intervention or control group and outcomes will be measured from birth and up to 4 months after delivery. The control group will receive standard counselling from Baby Friendly Hospitals, while the intervention group will receive the new EBF counselling intervention for overweight women. The intervention will be carried out at three time-points:

i) Last month of pregnancy.
ii) 24 h postpartum.
iii) Early infancy (1 and 3 months postpartum).

### Hypotheses and outcome variables

#### Primary hypotheses

Compared to standard management, the implementation of a new EBF counselling intervention for overweight woman will result in:

i) an increase in the prevalence of breastfeeding and EBF from birth up to four months of age;
ii) slower infant weight for length growth from birth up to four months of age;
iii) an increase in weight loss after delivery up to four months postpartum.

#### Primary outcomes and variables

Outcomes will be measured at birth (baseline) and 1, 3 and 4 months postpartum in the intervention and control groups. The primary outcomes are:

i) Prevalence of breastfeeding and EBF, ascertained by asking the mother about infant feeding practices during the last 24 h;
ii) Growth velocity from birth, defined as change in weight for length in g/cm and length for age in cm/days;
iii) Maternal weight loss after delivery in kg, using maternal weight at 24 h as baseline.

#### Secondary hypotheses

Compared to standard management, the implementation of a new EBF counselling intervention designed to support overweight woman will result in:

i) An increase in the volume of breast milk in ml as a result of an increase in serum prolactin levels in ng/ml, at 1 and 4 months.
ii) Higher milk prolactin concentration and adequate macronutrient content of protein, lactose, fat and energy, at 1 and 4 months, which will in turn be associated with more optimal infant growth (based on WHO growth standards).

### Secondary outcomes and variables

i) Maternal serum levels of prolactin and breast milk prolactin concentration and macronutrient content, measured at 1 and 4 months after delivery;
ii) Estimated breast milk intake at 1 and 4 months according to WHO recommendations [53].

**Fig. 1:**
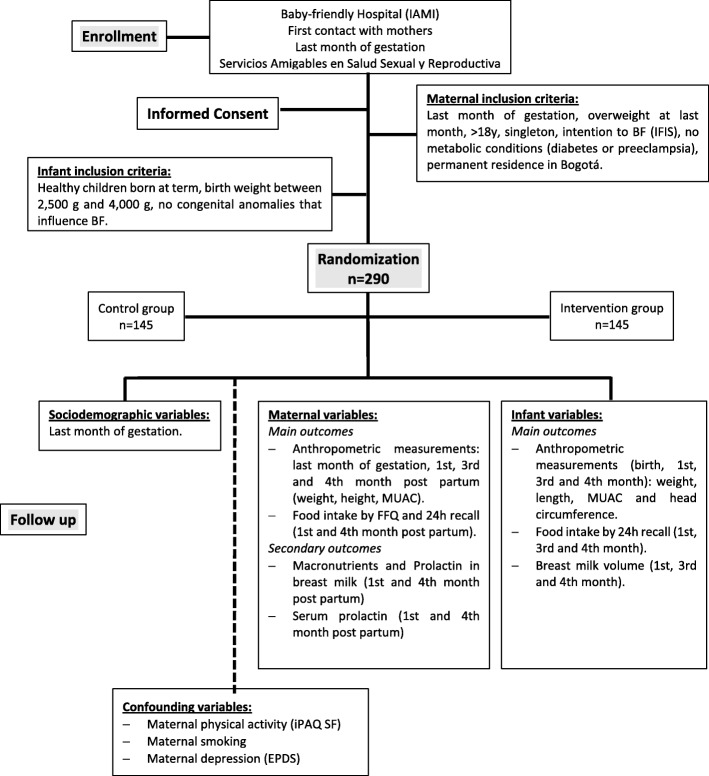
Study design to evaluate the effect of a new EBF counselling intervention in overweight women. Abbreviations: Infant Feeding Intention Scale (IFIS), Middle Upper Arm Circumference (MUAC), Food Frequency Questionnaire (FFQ), International Physical Activity Questionnaire Short Version (IPAQ SV), Edinburgh Postnatal Depression Scale (EPDS)

**Fig. 2:**
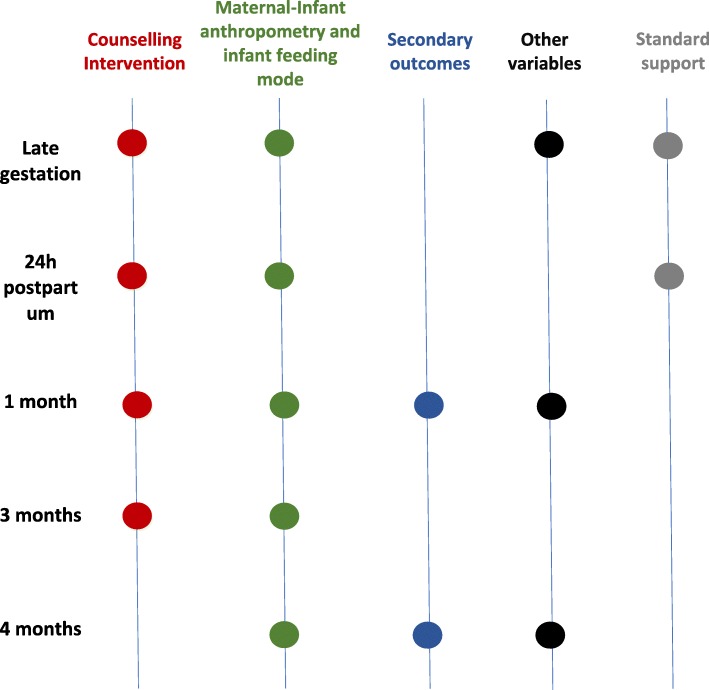
Timeline measurements and breastfeeding support for intervention and control groups. Secondary outcomes include breast milk sample and maternal serum sample. Other variables include maternal physical activity (IPAQ SV) and maternal food intake

The study design is shown in Fig. 1 and a timeline of measurements in Fig. 2.

### Study population

Pregnant women in the last trimester of gestation who meet the inclusion criteria and provide written informed consent (for vulnerable populations) will be recruited from the pregnant care program (CAPS) in a Baby Friendly Hospital in Bogotá, Colombia; the hospital is a referral institution for maternal and child care, where the prevalence of overweight pregnant women is 34.6%. The prevalence of obesity in children under 5 years in 2010 using BMI/age was 5.2% in Colombia and 6.4% in Bogotá. The hospital reported a population coverage of 1,620,000 for December 2016, and a total of 591 pregnant women in the program of Friendly Services in Sexual and Reproductive Health with a high proportion in the lowest socioeconomic groups.

The inclusion criteria will be:

*Women:* singleton pregnancy, overweight for gestational age (defined as BMI/GE ≥ 28.1 kg/m^2^ using Atalah’s criteria) measured at the 32nd week of gestation, older than 18 years, no pre-eclampsia or diabetes, permanent residence in Bogotá and intention to breastfeed. Women with hypertension during labour who do not require admission to the intensive care unit will be included in the study. Women with a history of breast surgery, maternal intensive care stay, postpartum depression risk at baseline (late pregnancy) based on the Edinburgh Postnatal Depression Scale (EPDS) [54], infection with human immunodeficiency virus (HIV) or treatment with cytotoxic drugs, cardiovascular medications, anticoagulants or psychopharmaceuticals will be excluded.

*Infant*: healthy term newborns (≥ 37 weeks), birth weight ≥ 2500–4000 g without any condition that interferes with breastfeeding practice such as galactosemia, cleft lip and/or cleft palate and congenital heart defect or disease.

### Sample size

The sample size was calculated to detect a difference in the prevalence of EBF between the intervention and control group of 23%, based on literature [43] reporting a baseline EBF of 22.8% in the control group (the prevalence of EBF for the Colombian population at 4 months in urban areas in 2010 [55]; there is a lack of data on EBF prevalence in overweight women in Colombia), with a power of 95%, an alpha error of 5% and a two-tailed calculation determined using the EpiInfo StatCalc program [56]. Assuming follow-up losses of 20%, 290 woman-child dyads are required, 145 in each group.

A convenience subsample of 60 women will be recruited, 30 from each group (20% of overall sample) for analysis of serum and milk prolactin concentration and macronutrient content.

### Recruitment

Overweight pregnant women (using Atalah’s criteria: BMI/GE ≥ 28.1 kg/m^2^) [57] will be contacted at 32 weeks of gestation at their hospital antenatal check-up. They will receive information about the study; those who agree to participate will be asked to provide written informed consent. To protect the identity of the women, they will be assigned an identification or ID number. Assistance with travel expenses for each visit to the clinic will be provided, and at the end of the study they will receive a gift to thank them for their participation in the study.

#### Randomization and masking

Each overweight woman will be assigned randomly to one group, intervention (breastfeeding counselling intervention) or control (standard counselling). Randomization assignments will be prepared by a member of the team who has no contact with the study subjects, using randomized blocks. Assignments will be stored in sealed opaque envelopes. It will not be possible to blind the researcher performing the intervention and collecting data, although laboratory measurements will be blinded. Mothers will be aware that the study involves assignment to one of two types of breastfeeding support but they will not know the details of this support. They will have the possibility to withdraw from the study at any time. To avoid contamination between groups, appointments will be scheduled individually by telephone contact.

#### Data collection

Data will be collected from all participants at five study time-points (last month of pregnancy, 24 h postpartum, and 1, 3 and 4 months postpartum) as described in Table 1. Questionnaires and assessments (detailed description below) will be completed at study visits as described in Figs. 1 and 2. The main information will be: i) sociodemographic and maternal and paternal health status questionnaire (last month of pregnancy); ii) maternal food consumption pattern measured using a maternal 24 h dietary recall [58, 59] and a semiquantitative food frequency questionnaire adapted for foods available in Colombia in a previous pilot study as described below; iii) maternal intention to breastfeed using the Infant Feeding Intention Scale adapted from the Nommsen-Rivers scale [60] (last month of pregnancy); iv) maternal risk of postpartum depression using the EPDS [54] (late gestation and 1 month postpartum); v) maternal physical activity using the IPAQ short version [61]; and vi) mother and infant anthropometric measurements. Breast milk macronutrient content will be determined at 1 and 4 months postpartum using infrared transmission spectroscopy (MIRIS Human Milk Analyzer) and serum and milk prolactin concentration will be determined by in vitro ELISA commercial kit.

**Table 1:** Study variables and data collection questionnaires to be used in the study

Time-point	Questionnaire or assessment	Description
Last month of gestation	General information	Name, address, phone number, maternal ID, maternal date of birth, health social scheme.
	Maternal socioeconomic and demographic information	Marital status, socioeconomic level, type of housing, maternal occupation, monthly income, educational level, maternal leave, income assigned to food purchase, access to tap water, person in charge to infant, breastfeeding family support.
	Pregnancy information	Maternal smoking, obstetric background, gestational age, probable date of birth, supplement and vitamins intakes, laboratory reports, medical history.
	Paternal information	Educational level, monthly income, weight and height (self-reported), medical history.
	Intention to breastfeed	Five questions about intention to breastfeed or give formula until 6 months postpartum.
	Maternal anthropometry	Pregestational weight (self-reported), weight, height and MUAC measurements.
	Breast examination	Nipple and breast self-perception.
	Counselling assessment	Detected problems and concerted solutions.
	IPAQ SV	Physical activity registry: type of activity, time (minutes per day), number of days per week.
	EPDS	Ten questions to evaluate maternal emotional status.
	Maternal 24-h dietary recall	Five steps 24HR: fast list, frequently forgotten foods, meals and time, food consumption in the last 24 h within detailed registry and standardized recipes.
	Semi-quantitative food frequency intake	Diary, weekly or monthly intake of 107 foods included in 8 food groups.
24 h postpartum	Maternal anthropometry Information about delivery and newborn	Weight and MUAC. Gender, date of birth, type of delivery, complications, gestational birth age, APGAR at 5 min, time of birth.
	Newborn anthropometry	Birth weight, length, head circumference, MUAC.
	Breastfeeding information	Time of skin to skin contact, duration of skin to skin contact, breastfeeding during the first hour after delivery, breastfeeding difficulties.
	Infant 24-h recall	Time, place, infant feeding in the last 24 h (including breastfeeding, infant formula or other type of foods given to the baby).
	Breast milk intake	Number of breastfeeds per day and night, time per each breastfeed, appetite (mother’s perception).
	Breast latching	Observation of latching, swallowing, type of nipple, maternal comfort perception and breastfeeding position.
	Counselling registry at 24 h, 3th day, 7th day and 14th day	Detected problems and agreed solutions, success of breastfeeding counselling regarding the last meeting’s concerted solutions.
1 month postpartum	Maternal anthropometry	Weight and MUAC.
	Infant anthropometry	Weight, length, head circumference, MUAC.
	Counselling registry	Detected problems and concerted solutions, success of breastfeeding counselling regarding the last meeting’s concerted solutions.
	IPAQ SV	Physical activity registry: type of activity, time (minutes per day), number of days per week.
	EPDS	Ten questions to evaluate maternal emotional status.
	Maternal 24-h recall	Five steps 24HR: fast list, frequently forgotten foods, meals and time, food consumption in the last 24 h within detailed registry and standardized recipes.
	Semi-quantitative food frequency intake	Diary, weekly or monthly intake of 107 foods, included in 8 food groups.
	Infant 24-h recall	Time, place, infant feeding in the last 24 h (including breastfeeding, infant formula or other type of foods given to the baby).
	Breast milk intake	Number of breastfeeds per day and night, time per each breastfeeds, appetite (mother’s perception).
	Breast milk macronutrient content	Protein, lactose and fat content in human milk
	Prolactin in maternal serum	Concentration of prolactin hormone in maternal serum, 2 h after maternal wake up
3 months postpartum	Maternal anthropometry	Weight and MUAC.
	Infant anthropometry	Weight, length, head circumference, MUAC.
	Breast examination	Nipple and breast observation.
	Counselling registry	Detected problems and concerted solutions, applicability of last meeting’s concerted solution
	Infant 24-h recall	Time, place, type of food.
	Breast milk intake	Number of breastfeeds per day and night, time per each breastfeed, appetite (mother’s perception).
	Macronutrients content in breast milk	Protein, lactose and fat content in human milk.
	Prolactin in maternal serum	Concentration of prolactin hormone in maternal serum, 2 h after maternal wake up.
4 months postpartum	Maternal anthropometry	Weight and MUAC.
	Infant anthropometry	Weight, length, head circumference, MUAC.
	Breast examination	Nipple and breast observation.
	Assessment of breastfeeding counselling	Successful of new approach in EBF counselling with 5 questions to assess concerted solutions.
	IPAQ SV	Physical activity registry: type of activity, time (minutes per day), number of days per week.
	Maternal 24-h recall	Five steps 24HR: fast list, frequently forgotten foods, meals and time, food consumption in the last 24 h within detailed registry and standardized recipes.
	Semi-quantitative food frequency intake	Diary, weekly or monthly intake of 107 foods, included in 8 food groups.
	Infant 24-h recall	Time, place, infant feeding in the last 24 h (including breastfeeding, infant formula or other type of foods given to the baby).
	Breast milk intake	Number of breastfeeds per day and night, time per each breastfeed, appetite (mother’s perception).

### Breastfeeding counselling intervention

A new approach towards EBF counselling was designed based on Rogers’ Client-Centred Theory. The women assigned to the intervention group will receive the intervention at three key time-points: i) last month of gestation to prepare and promote the importance of early contact and breastfeeding initiation in the first hour postpartum, and to prepare the women for difficulties they may encounter in establishing breastfeeding; ii) 24 h postpartum to ensure that EBF is being established and to identify any difficulties the mother is experiencing; iii) early infancy (1 and 3 months postpartum) to identify breastfeeding problems and empower the women to continue exclusive breastfeeding. The intervention will be conducted by a certified breastfeeding counsellor with listening skills and an understanding of the particular problems faced by overweight women during breastfeeding, who will analyse the environment and maternal breastfeeding problems in order to reach consensus solutions with the woman.

#### New EBF counselling intervention

For the purpose of this study, EBF counselling for overweight women is defined as a well-structured and permissive relationship built on trust between the counsellor and the pregnant and breastfeeding woman, using her feelings, beliefs and sociocultural environment to gain an understanding of herself and her situation in order to empower the woman and achieve EBF for six months.

#### Intervention theoretical framework

Based on Carl Rogers’ Client-Centred Theory [62], this intervention will have three main principles: i) dialogue between counsellor and woman in an atmosphere of respect for her individuality and particular situation related to breastfeeding; ii) empowerment of the woman, raising awareness to allow her to choose the best alternative to achieve her goals for EBF; and iii) concerted solutions under the guidance of the counsellor who will give a set of alternatives to solve problems related to breastfeeding at critical moments, which will be mainly, but not exclusively, mechanical problems and/or technical problems with breast milk production.

#### Essential time-points for intervention

Three key time-points to intervene were identified to increase the duration of EBF in overweight women, according to their particular needs [63]: i) the last month of gestation as the moment to identify mechanical problems such as larger breasts and flattened nipples [64] and the intentions and fears of the women regarding breastfeeding [65], to promote the importance of early contact (skin to skin) between the women-infant dyad [66] and initiation of breastfeeding in the first hour postpartum [67], and to prepare the women to recognize adequate infant latch-on; ii) 24 h postpartum to evaluate suckling, early initiation of breastfeeding and beginning of lactogenesis II [68]; iii) early infancy (1 and 3 months postpartum) to empower the women in the continuation of EBF (periods where there is a risk of early weaning) [69]. During each counselling session, counselling support materials with key messages will be given to the women to reinforce counselling. Counselling will be evaluated at every time-point using a structured questionnaire about maternal satisfaction and usefulness. Adherence to EBF will be assessed by asking the mother if she has exclusively breastfed during the last 24 h at each time-point.

Women with breastfeeding difficulties at the time of the intervention that are beyond the scope of the researcher’s competence will be referred to the relevant professional so that appropriate treatment can be offered to promote continued breastfeeding (e.g. physician, nurse, psychologist). Referrals and the reason for the consultation will be recorded and compared between groups.

#### Standard counselling

This will be performed in the control group, based on the institutional and national policy for breastfeeding (Resolution 412 of the year 2000). During pregnancy, women receive a group talk about the importance of breastfeeding as part of the maternity preparation course at the institution. During labour, early contact and early initiation of breastfeeding is supported by health workers at the institution who identify the risks for the woman-child dyad. During hospital discharge, women receive nutritional recommendations with an emphasis on EBF during the first 6 months. During the first 4 months postpartum, the follow-up time of the study, it is estimated that women in the control group will attend two follow-up growth and development visits, where the weight and length of the child is evaluated and breastfeeding support is given by group activities or individual consultation carried out by health professionals. The intervention group will also receive this standard follow up, as well as the new approach in EBF counselling. Table 2 summarizes the main characteristics of the new approach compared with the standard counselling given by the institution.

**Table 2:** Time-points and description of breastfeeding counselling among control and intervention groups

Time-point	Intervention group		Control group	
	Issues to follow	Responsible	Standard care	Responsible
Last month of gestation	1. Dialogue with the women to determine risk of possible breastfeeding problems: - Fears and expectations - Breast examination - Breastfeeding problems - Intention to breastfeed 2. Maternal empowerment - Knowledge of familiar and social environment - Motivation - Early contact (skin to skin contact) - Commitment 3. Agreed solutions - Individuality respect - Realistic and assertive solutions	Researcher	Information on the benefits of breastfeeding and breastfeeding positions through a one-hour group talk as part of the institution’s course on preparation for motherhood.	Nursing Department
24 h postpartum	1. Dialogue with the women to evaluate problems and difficulties identified at gestation: - Fears and expectations - Early breastfeeding - Skin to skin contact - Adequate latching and suckling - Human milk volume 2. Maternal empowerment - Breastfeeding crisis: fatigue, fears and worries. - Motivation 3. Agreed solutions - Respect for individuality - Realistic and assertive solutions.	Researcher	Institutional policy to allow rooming in, skin to skin contact. Risks of early weaning. Nutritional recommendations about breastfeeding importance at hospital discharge.	Institutional health care professionals
1 month postpartum	1. Dialogue with the women to follow up the agreed solutions and identify new difficulties with breastfeeding - Maternal perception of milk volume produced - Maternal perception of infant appetite 2. Maternal empowerment - Fears and expectations: low breast milk volume - Motivation and solutions follow up - Self-esteem 3. Agreed solutions - Individuality respect - Solutions follow up and measure of breastfeeding achievements	Researcher	Six visits during the first 12 months to follow up infant growth and development. It includes education in breastfeeding in group meetings. Breastfeeding counselling on demand or by referral.	Institutional health care professionals: Nurse Department
3 months postpartum	1. Dialogue with the women to follow up agreed solutions and identify new breastfeeding difficulties: - Back to work, study and other activities. - Early initiation of complementary food 2. Maternal empowerment - Fears and expectations - Motivation and follow up of solutions - Self-esteem 3. Agreed solutions - Individuality respect - Solutions follow up and measure of breastfeeding achievements	Researcher	Six visits during the first 12mo to follow up infant growth and development. Lack of adherence to the program.	Institutional health care professionals, Prevention and promotion programs at the institution.

#### Measures

Questionnaires and assessments will be used to collect information among intervention and control groups at specific time-points, as shown in Table 1.

- Maternal socioeconomic and demographic information, pregnancy information and paternal information: Information about residency, age, marital status, occupation, socioeconomic level, family size, family income, educational level, maternity leave, access to public services, social security, health benefits, smoking, alcohol consumption, family and medical history will be recorded. Gestational age will be determined by the date of the last menstruation, corroborated by sonographic studies available in the clinical notes. Paternal information will be reported by the woman and will include health status and monthly income.
- Intention to breastfeed: The scale uses five simple questions to determine the method of feeding that the woman plans for the baby. The scale has been validated and translated in previous studies [60].
- Maternal anthropometry: Anthropometric data - weight, height and mid upper arm circumference (MUAC) - will be collected in the last month of pregnancy, 24 h postpartum, and 1, 3 and 4 h months postpartum in minimal clothes. Weight will be measured in duplicate using an electronic scale (SECA 813, capacity 200 kg, precision 100 g), height measured in triplicate using a portable stadiometer (SECA 213, up to 205 cm) and MUAC measured in triplicate to the nearest cm (SECA 203 measuring tape, range 0 to 205 cm, graduation 1 mm). Weight gain during gestation (self-reported weight at the end of gestation minus pre-gestational weight) will be used as a baseline characteristic and as a covariate to predict the main outcomes.
- Breast examination: Participants will complete a questionnaire using illustrations of breasts to identify possible anatomical aspects that could make breastfeeding difficult (e.g. large breasts, flat nipples). This information will be used to focus the intervention for individual mothers.
- Edinburgh Postnatal Depression Scale (EPDS): This consists of 10 questions about feelings during the last week. It will be administered during the last month of gestation and 1 month postpartum as a tool to determine the risk of postpartum depression [54].
- Counselling register and evaluation: To assess the content of and adherence to the new EBF counselling intervention, a record of the problems identified will be kept during each visit. A questionnaire will also be administered by an independent investigator blinded to group allocation at the end of the study period (four months postpartum) which will include information on the problems identified, solutions and success, measured as: not successful (no problem resolution); limited success (partial resolution of the problem), or successful (problem solved).
- IPAQ short version: Physical activity questionnaire which considers types of physical activity, time (minutes per day) and number of days per week, and number of hours per day that the woman remains seated [61].
- Maternal food consumption: Determined using a standard food frequency questionnaire used and adapted in Colombian populations in other studies, and two multiple steps 24-h recall questionnaires to determine food consumption and nutrient intake including supplements, vitamins, minerals and maternal feeding problems; to be used as a baseline descriptive characteristic and as a potential confounder. This methodology is based on that described by the United States Department of Agriculture [58, 59]. To improve the quality of the data, photographs will be used to determine portion size [70]. Data analysis will be conducted using Colombian food composition tables [71], USDA standard nutrient reference database and food labels.
- Delivery information: Data about the type of delivery, newborn APGAR score, time of breastfeedng initiation, maternal-infant complications including those which delay the initiation of breastfeeding, and drugs administered during labour and the first 4 months after delivery.
- Newborn and infant anthropometry: Anthropometric variables - weight, length, MUAC and head circumference - will be measured by a trained nutritionist and dietitian following WHO protocol [72] at birth and 1, 3 and 4 months postpartum. Infant weight will be measured in duplicate using an electronic baby scale (TANITA 1583 with precision of 10 g, capacity 200 kg), length measured in triplicate using an infantometer (SECA 417, folding mechanism and foot stop, measuring range up to 100 cm), and head circumference and MUAC measured using a pediatric tape measure (SECA 201, range 0 to 205 cm, graduation 1 mm). Infant growth velocity will be defined as the change in the Z-score of weight for length, length for age, head circumference for age and mid upper arm circumference for age from birth to four months according to WHO growth standards [73]. Data collection will be performed by the same researcher in both groups to control information bias among observers.
- Breastfeeding information: This questionnaire collects information about initiation of breastfeeding at the hospital.
- Infant 24-h dietary recall: To determine the EBF prevalence, an infant 24-h recall will be used asking mothers about the foods consumed by the child during the previous day and infant feeding practices at 1, 3 and 4 months. = The counsellor will ask about the initiation, frequency and mode of preparation of specific beverages and semisolid foods or supplements since the last visit. This methodology will allow breastfeeding to be classified as exclusive or predominant according to WHO definitions [12].
- Breast-latching observation: Considers information about position of breastfeeding and maternal-infant perception of comfort during breastfeeding. This is a check-list that the investigator completes after watching a breastfeed [74].
- Breast milk macronutrients: Expression of breast milk (fore milk) will be performed at 1 and 4 months postpartum at the researcher’s office in the morning, using a Philips Single Electric Breast Pump under researcher supervision. The sample will be stored at − 20 °C in the laboratory at the Pontificia Universidad Javeriana for analysis. Content of macronutrients (carbohydrate, protein and fat) and energy in breast milk will be determined using the MIRIS Human Milk Analyzer, which uses infrared transmission spectroscopy and requires a human milk sample of 5 ml. Results will be recorded in the database.
- Breast milk prolactin: Determination of breast milk prolactin concentration will be performed using an Abcam ab226901 Human Prolactin SimpleStep ELISA kit. The procedure requires 2 ml of breast milk.
- Serum prolactin: To determine the maternal serum prolactin concentration, a non-probabilistic convenience subsample of 60 (20%) women will be selected. The blood sample will be taken at the laboratory of the institution, within 2 h of waking in the morning and before any food is consumed. The procedure will require 5 ml of maternal blood, which will be analyzed by ELISA (Abcam ab226901).

All questionnaires and assessments will be administered by the researcher.

#### Pilot study

Due to the lack of information about characteristics of overweight pregnant women in Bogotá, a pilot study was performed among pregnant women (*n* = 209) to determine maternal socioeconomic characteristics, anthropometric status, prenatal health status, intention to breastfed, physical activity and eating patterns. Briefly, 33.7% of participants were overweight in the last trimester of pregnancy, 23.7% gained more than 13 kg by the last trimester and among overweight women, 37.2 and 31.3% had a strong and very strong intention to breastfeed. The pilot study showed the prevalence of overweight among pregnant women in the planned study population was similar to that reported previously [55]. There was also a very high intention to breastfeed among overweight women, justifying the plan to develop and test a counselling intervention.

The pilot study was also an opportunity to test the questionnaires and validated scales (such as the intention to breastfeed scale) and to evaluate the time needed to complete the questionnaires. Following the pilot study, questions about breastfeeding practice were also changed to improve the maternal understanding of the questions.

#### Data management

To guarantee the quality of the data, four checks will be performed: i) double checking of the information recorded in the questionnaires; ii) breastfeeding support verification (questionnaire administered by an independent investigator) in all mothers who completed the follow up; iii) random selection of questionnaires to check the consistency between data entered in the database and questionnaires; and iv) preliminary database check to identify extreme data and missing data using IBM SPSS Statistics software version 24. The questionnaires containing the original information will be stored safely at the Pontificia Universidad Javeriana for five years and the digital database will be stored as a computer file with access code. Clinical visits for breastfeeding counselling will be scheduled on different days for control and intervention groups to avoid contamination bias.

#### Statistical analysis

Initially, a descriptive analysis of the variables will be performed to characterize the sample and to determine the comparability between the intervention and control groups. A bivariate analysis will be performed to identify significant associations between the prevalence of EBF, growth velocity and maternal weight loss; the normality of the data distribution and the homogeneity of the variance will be checked to determine the appropriate parametric or non-parametric test for analysis. The main analysis will be performed on an intention-to-treat (ITT) basis. Analysis of variance and a random effects model will be performed to determine differences in EBF prevalence, infant growth velocity and maternal weight loss between groups. A sub-group analysis to compare differences between control and intervention groups will be carried out by multiple regression model, whether the outcome is continuous, binary or survival time. IBM SPSS Statistics version 24 will be used for data analysis.

#### Ethical issues

The research project is approved by the Ethical Committee at the Faculty of Sciences at the Pontificia Universidad Javeriana and the Ethical Committee at the institution selected for the study (Code Approval SNCI-021-CEI Acta 08, 28 April 2018). Each participating woman in the study will receive detailed information about the study and will have the opportunity to ask questions about the research. Those women who agree to participate will be included in the study after reading and signing the written informed consent form. Women will have the right to withdraw from the study at any time, without affecting their care in the institution where the project is conducted. All mothers will receive assistance with travel expenses for each visit to the clinic, and at the end of the follow up they will receive a token gift (estimated value USD$4) to thank them for their participation in the study.

According to Resolución 8340 of 1993 of the Colombian government, the present study uses an intervention with minimum risk and represents a low health risk for the women related to venous blood samples for the serum prolactin sample or breast milk expression. The protocol requires the woman and two witnesses to sign the written informed consent for vulnerable populations and they will provide consent for the analysis and publication of their data for scientific purposes.

## Discussion

WHO recommends that infants be exclusively breastfed until 6 months with continued breastfeeding alongside complementary feeding, to prevent infectious diseases and potentially reduce the risk of later obesity. Infants of mothers who are overweight or obese during pregnancy are at increased risk of obesity and more breastfeeding might lower this risk. However, breastfeeding is often challenging for overweight women as they face emotional, psychosocial, and hormonal and physiological issues that reduce both initiation and duration. There is a lack of research in overweight women on interventions to increase breastfeeding, including the effects of breastfeeding counselling, and in Colombia there is currently no specific strategy to support overweight women to achieve their breastfeeding goals.

Given the increasing prevalence of obesity worldwide, especially in children and women of childbearing age, combined with lower breastfeeding rates in overweight women, there is an urgent need to design, develop, validate, implement and disseminate efficient strategies for promoting breastfeeding in this population. This study aims to provide the scientific evidence which might support public policies aimed to implement programs and low delivery cost strategies for EBF counselling in overweight women.

### Strengths and limitations

The planned study has a number of strengths, notably the experimental design, implementation of a new counselling approach to help overweight mothers to breastfeed more successfully, and assessment of the effect of the intervention on breastfeeding outcomes but also on infant growth and maternal weight loss. However, the study also has some limitations. First, estimation of breast milk volume will be carried out using an algorithm because it is not considered feasible to use the gold standard (deuterium dilution) method in the study population. Second, serum and breast milk prolactin will be measured in a subsample due to resource limitations. Finally, for logistic reasons it is not possible to carry out the study in several health institutions covering all socioeconomic levels which may have implications for the generalisability of the results.

## Data Availability

Not applicable.

## Abbreviations

BMI: Body mass index
EBF: Exclusive breastfeeding
EPDS: Edinburgh Postnatal Depression Scale
FFQ: Food Frequency Questionnaire
IFIS: Infant Feeding Intention Scale
IPAQ SV: International Physical Activity Questionnaire Short Version
ITT: Intention to treat
MUAC: Middle upper arm circumference
WHO: World Health Organization
